# Bridging oxidase catalysis and oxygen reduction electrocatalysis by model single-atom catalysts

**DOI:** 10.1093/nsr/nwac022

**Published:** 2022-02-23

**Authors:** Xiangyu Lu, Shanshan Gao, Han Lin, Han Tian, Deliang Xu, Jianlin Shi

**Affiliations:** State Key Laboratory of High-Performance Ceramics and Superfine Microstructure, Shanghai Institute of Ceramics, Chinese Academy of Sciences, Shanghai 200050, China; Center of Materials Science and Optoelectronics Engineering, University of Chinese Academy of Sciences, Beijing 100049, China; Shanghai Tenth People's Hospital, Shanghai Frontiers Science Center of Nanocatalytic Medicine, The Institute for Biomedical Engineering and Nano Science, School of Medicine, Tongji University, Shanghai 200092, China; School of Public Health, Shanghai Jiao Tong University School of Medicine, Shanghai 200025, China; State Key Laboratory of High-Performance Ceramics and Superfine Microstructure, Shanghai Institute of Ceramics, Chinese Academy of Sciences, Shanghai 200050, China; Shanghai Tenth People's Hospital, Shanghai Frontiers Science Center of Nanocatalytic Medicine, The Institute for Biomedical Engineering and Nano Science, School of Medicine, Tongji University, Shanghai 200092, China; State Key Laboratory of High-Performance Ceramics and Superfine Microstructure, Shanghai Institute of Ceramics, Chinese Academy of Sciences, Shanghai 200050, China; Center of Materials Science and Optoelectronics Engineering, University of Chinese Academy of Sciences, Beijing 100049, China; State Key Laboratory of High-Performance Ceramics and Superfine Microstructure, Shanghai Institute of Ceramics, Chinese Academy of Sciences, Shanghai 200050, China; State Key Laboratory of High-Performance Ceramics and Superfine Microstructure, Shanghai Institute of Ceramics, Chinese Academy of Sciences, Shanghai 200050, China; Center of Materials Science and Optoelectronics Engineering, University of Chinese Academy of Sciences, Beijing 100049, China; Shanghai Tenth People's Hospital, Shanghai Frontiers Science Center of Nanocatalytic Medicine, The Institute for Biomedical Engineering and Nano Science, School of Medicine, Tongji University, Shanghai 200092, China

**Keywords:** nanocatalytic medicine, tumour therapy, oxygen reduction electrocatalysis, oxidase, single-atom catalysts

## Abstract

Nanocatalysts with enzyme-like catalytic activities, such as oxidase mimics, are extensively used in biomedicine and environmental treatment. Searching for enzyme-like nanomaterials, clarifying the origins of catalytic activity and developing activity assessment methodologies are therefore of great significance. Here, we report that oxidase catalysis and oxygen reduction reaction (ORR) electrocatalysis can be well bridged based on their identical activity origins, which makes facile electrocatalytic ORR activity measurements intrinsically applicable to oxidase-like activity evaluations. Inspired by natural heme-copper oxidases, Cu/Fe-doped single-atom catalysts (SACs) were first synthesized and used as model catalysts. Chromogenic reactions, electrochemical voltammetric measurements and density functional theory calculations further verified the linear relationship between the oxidase-like and ORR catalytic activities of the catalysts; thus, an effective descriptor ($| {\overline {{j_{\rm{n}}}} } |$) is proposed for rapid enzymatic catalyst evaluation. Evidence suggests that the enhanced tumour therapeutic efficacy of SACs is a result of their oxidase-like/ORR activities, which proves that numerous ORR electrocatalysts are promising candidates for oxidase mimics and tumour therapy. The synergistic catalytic effect of the biomimetic heterobinuclear Cu-Fe centres has also been thoroughly probed.

## Teaser

Oxidase catalysis and oxygen reduction reaction (ORR) electrocatalysis are revealed to be homologous, which results in a linearpositive correlation between these two catalytic activities, and the electrochemical methodologies are introduced to search oxidase mimics for tumour therapy.

## INTRODUCTION

Nanomaterials with intrinsic enzyme-like characteristics show broad application potential (for example, in biosensors, immunoassays, disease diagnosis, medicine development, antifouling and environmental treatment) owing to their high stability, low cost, tunability and high catalytic activity [1–4]. Among them, oxidases can catalyse the oxidation of organic molecules using O_2_, a process during which O_2_ is concurrently reduced to water, hydrogen peroxide or functional groups [5–8]. Catalysis of the oxidation of biomolecules by oxidizing species such as O_2_ and H_2_O_2_ forms the basis of diverse biomedical applications, such as tumour therapy and antibiosis [9–11]. Unfortunately, H_2_O_2_ is generated from O_2_ in a kinetically slow manner in the preceding metabolic processes; thus, its *in vivo* level is usually below a certain threshold, so the therapeutic outcomes of using H_2_O_2_ as a substrate are undoubtedly limited [12,13]. Based on the principle of direct matter conversion by using O_2_ as the reaction substrate, oxidases are the most promising candidates for increasing oxidative stress owing to the sustainable supply of O_2_ via blood circulation and their higher utilization efficiency with regard to O_2_ than H_2_O_2_-involved pathways. Recent studies have found oxidase-like properties of a number of electrocatalysts that have been used in oxygen reduction reactions (ORRs) [9,14], while some natural oxidases have also been explored as oxygen reduction electrocatalysts [15–17]. Therefore, we hypothesize that the oxidase-like activities of catalysts are in accordance with their ORR activities in terms of their homology in activity origins. If this hypothesis is tenable, then we could efficiently assess the oxidase-like activity of catalysts based on mature and rather facile voltammetric techniques, and great numbers of known oxygen reduction electrocatalysts, which have been widely employed in fuel cells, dye-sensitized solar cells and metal-air batteries [18,19], may have oxidase-like activities and could be further applied in other fields.

To verify this hypothesis, single-atom catalysts (SACs), in which single metal atoms as catalytically active sites are individually dispersed and constrained on a support, were chosen as model catalysts. SACs are ideal heterogeneous catalysts featuring maximized utilization of metal atoms while minimizing ion cytotoxicity without free-metal-ion release into the media [20]. Additionally, the well-defined and uniform structure of the catalytic site provides an ideal model system for obtaining an overall understanding of the reaction mechanisms at the molecular level [21–24]. More importantly, the catalytic activity of SACs clearly originates from the active metal atom centres, so it does not depend on the size or surface characteristics of the support. Hence, we believe that mechanistic studies based on SACs will be of general significance.

**Figure 1.**
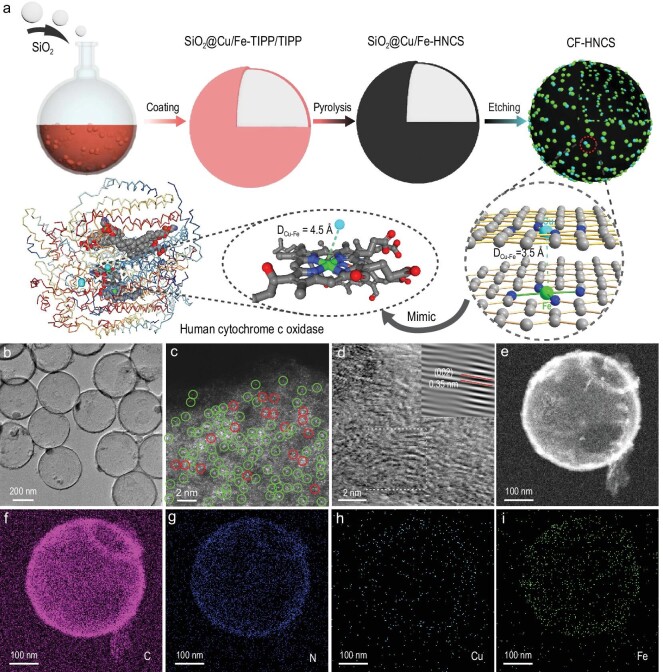
CF-HNCS synthesis and morphological characterization. (a) Procedure to synthesize CF-HNCS. (b) TEM image. (c) AC HAADF-STEM image. Bright single dots are marked by green circles and dual dots are marked by red circles. (d) HR-TEM image and corresponding FFT pattern (inset) of the marked area of CF-HNCS. (e) HAADF-STEM image and (f–i) corresponding EDS mappings of CF-HNCS: (f) C, (g) N, (h) Cu and (i) Fe.

In nature, heme-copper oxidases (HCOs), such as cytochrome c oxidases (Fig. 1a) containing a heterobinuclear Cu–Fe centre, can efficiently catalyse the four-electron reduction of dioxygen to water with minimal overpotential in the final step of respiration [25]. The chemical energy released in this reaction is subsequently stored in adenosine triphosphate (ATP), which is a common energy molecule in most forms of life [26]. As essential micronutrients, small amounts of copper and iron ions will have negligible side effects on tissues and organs [27,28].

Therefore, based on the above considerations, we have developed four types of model SACs with or without Cu and/or Fe ions constrained on a carbon matrix: (i) hollow N-doped carbon sphere (HNCS); (ii) single atoms of Cu constrained on HNCS (C-HNCS); (iii) single atoms of Fe constrained on HNCS (F-HNCS); and (iv) heterobinuclear Cu–Fe centres constrained on HNCS (CF-HNCS). In this study, by using these model SACs and employing chromogenic reactions, a voltammetric technique and density functional theory (DFT) calculations, we find that the oxidase-like and electrochemical ORR activities of the catalysts are homologous in nature and quantitatively show a good linear relationship with each other. Thus, a novel, facile and general oxidase-like activity assessment methodology based on the voltammetric technique could be well developed to rapidly screen thousands of potential catalysts as oxidase mimics. Furthermore, we demonstrate the synergistic effect of Cu–Fe biatoms in biomimetic CF-HNCS catalysts and their remarkably high catalytic activities in both organic oxidation and tumour therapy.

## RESULTS AND DISCUSSION

### Design, synthesis and characterization

Figure 1a illustrates the CF-HNCS synthesis procedure. Briefly, 5,10,15,20-tetra(4-(imidazol-1-yl)phenyl)porphyrindine (TIPP), Cu-TIPP, Fe-TIPP and *α*,*α*´-dibromo-p-xylene were subjected to quaternization reactions (Scheme S1) on the surface of prepared SiO_2_ nanospheres (Fig. S1a) to produce SiO_2_@Cu/Fe-TIPP/TIPP-polymer (Fig. S1b). Consequently, the SiO_2_@Cu/Fe-TIPP/TIPP-polymer was pyrolysed in a hydrogen/argon atmosphere. Moreover, Cu and/or Fe moieties within the polymer would be carbothermally reduced to form isolated single metallic Cu or Fe and bimetallic Cu–Fe sites coordinated with pyridinic N. Finally, the SiO_2_ core was etched off by NH_4_HF_2_, leading to CF-HNCS formation. As shown in Fig. 1b and Fig. S1c, CF-HNCS of ≈390 nm in diameter displays a uniform size distribution and maintain a spherical SiO_2_ morphology.

Figure 1c shows an aberration-corrected high-angle annular dark-field scanning transmission electron microscopy (AC HAADF-STEM) image of CF-HNCS. The bright single dots in green circles imply the possible existence of single Cu and Fe atoms because both Cu and Fe atoms are much heavier than the nitrogen-doped carbon support. A number of coupled atom pairs are marked with red circles. Although we tried to assign these possible double-atom pairs to dual Cu–Fe atoms, unfortunately we were not able to reach such a conclusion unambiguously because they could also be Cu–Cu or Fe–Fe dimers, and they may also be ascribed to metal atoms that are separated by height in the 3D carbon support but almost overlap in the 2D imaging projection. The high-resolution TEM (HR-TEM) image and fast Fourier transform (FFT) pattern show that a small part of the carbon matrix has crystallized, and a graphite layer with a d-spacing of ∼0.35 nm can be clearly observed, which can be attributed to the (002) lattice plane (Fig. 1d); this result corresponds to the X-ray diffraction (XRD) pattern (Fig. S2a) and the Raman spectra (Fig. S2b). The HAADF-STEM results and the corresponding energy-dispersive X-ray spectroscopy (EDS) mapping results demonstrate the uniform distributions of N, Cu and Fe in the carbon matrix (Fig. 1e–i).

### Fine structural characterization

**Figure 2.**
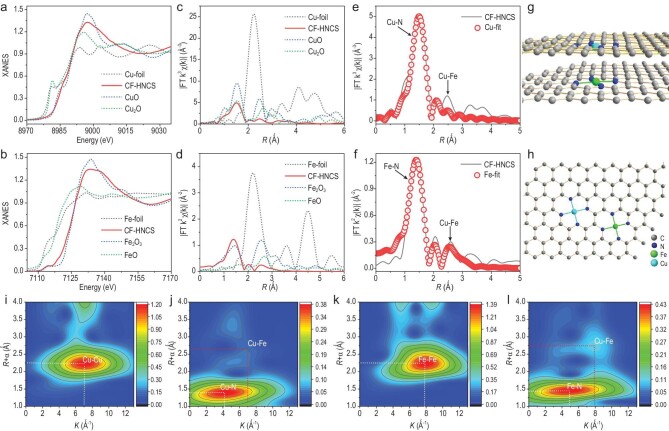
Fine structure characterization of the active sites. (a and b) XANES spectra at the (a) Cu K-edge and (b) Fe K-edge of CF-HNCS. (c and d) Fourier transforms at the (c) Cu K-edge and (d) Fe K-edge of CF-HNCS. (e and f) Corresponding (e) Cu K-edge and (f) Fe K-edge EXAFS fitting results of CF-HNCS in R space. (g and h) Proposed arrangement of Cu–Fe dual sites formed on neighbouring but opposite graphite layers ((g) CF-HNCS-1) and on the same carbon layer ((h) CF-HNCS-2). (i and j) WTs of the k^2^-weighted Cu K-edge EXAFS signals of (i) Cu foil and (j) CF-HNCS. (k and l) WTs of the k^2^-weighted Fe K-edge EXAFS signals of (k) Fe foil and (l) CF-HNCS.

To probe the chemical structures of the Cu and Fe atoms in depth, X-ray absorption spectroscopy (XAS) and X-ray photoelectron spectroscopy (XPS) were adopted. The Cu 2*p*_3/2_ and Fe 2*p*_3/2_ XPS spectra are shown in Fig. S3. The Cu peak is located at 934.9 eV, revealing the ionic Cu*^δ^*^+^ (*δ* ≈ 2) nature of Cu in CF-HNCS and C-HNCS [29,30]. As shown in the Cu K-edge X-ray absorption near edge spectroscopy (XANES) profiles, the solid red line intensity and pre-edge peaks of CF-HNCS and C-HNCS are located between those of Cu_2_O and CuO, revealing that the valence of Cu is between +1 and +2 and the oxidation state is closer to +2 (Fig. 2a and Fig. S4a). The Fe 2p spectrum (Fig. S3b) exhibits a major peak at 710.7 eV, suggesting that the metal species is in the oxidation states in CF-HNCS and F-HNCS [31]. Furthermore, the oxidation state of Fe ions in CF-HNCS and F-HNCS was determined to be between +2 and +3 from the Fe K-edge XANES profiles (Fig. 2b and Fig. S5a). To examine the local coordination environment of Cu and Fe sites, we investigated the extended X-ray absorption fine structure (EXAFS) of the SACs. Fourier transformed EXAFS (FT-EXAFS) analysis of the Cu K-edge in *R* space shows that the peaks at 2.22 Å (uncorrected) present in the Cu–Cu shell of Cu foil are absent in CF-HNCS and C-HNCS, implying that most Cu atoms are isolated (Fig. 2c and Fig. S4b–d). Similarly, the FT-EXAFS spectra of the Fe K-edge reveal that Fe atoms are also atomically dispersed in CF-HNCS and F-HNCS (Fig. 2d and Fig. S5b–d). However, Cu–Fe bonding probably exists in CF-HNCS according to the extracted accurate coordination numbers (CNs) and bond lengths, suggesting that some of the copper species chemically interact with the Fe species (Fig. 2e and f, and Tables S1 and S2). The CNs of Cu–Fe bonds in CF-HNCS samples are 0.9 from both Cu K-edge and Fe K-edge EXAFS fitting curves, confirming that the interacting metal atoms exist mainly in the form of bimetallic Cu–Fe atomic clusters in Cu–Fe–N–C rather than in the form of metal-based nanoparticles. The Cu–Fe bond length is ∼3 Å in CF-HNCS, which is close to the d-spacing of the graphite layer (3.5 Å) in the matrix. The CNs of Cu–N and Fe–N bonds in the first shell are similar and ∼4 Å, implying that similar Cu-N_4_ bonding and Fe-N_4_ bonding forms in the N-doped carbon matrix. In summary, each Cu or Fe atom is coordinated with four N atoms to form Cu-N_4_ or Fe-N_4_ sites in C-HNCS or F-HNCS, respectively (Figs S4f and S5f); while most metal atoms are isolated in the form of metal-N_4_, a small portion of the metal species are anchored on neighbouring graphite layers to form Cu–Fe dual atomic sites in CF-HNCS-1 (Fig. 2g). Because the Cu and Fe atoms anchored on the same carbon layer may also have synergetic interactions, we propose an arrangement of Fe–Cu dual atomic sites in CF-HNCS-2 (Fig. 2h), although this long-range interaction is hard to detect.

Furthermore, wavelet transform (WT) analysis was performed to verify the backscattering atoms. As illustrated in Fig. 2i and k, the first intensity maximum of Cu–Fe–N–C at 1.40 Å (uncorrected), correlated with a k-value of ∼4.2 Å^–1^, and that at 1.45 Å (uncorrected), correlated with a k-value of ∼4.9 Å^–1^, could be ascribed to Cu-N bonding and Fe–N bonding, respectively. Compared with the WT plots of Cu and Fe foils (Fig. 2i and k), the intensity maxima corresponding to Cu–Cu and Fe–Fe are absent, further confirming that there are no metal-based nanoparticles. The second intensity maximum of Cu–Fe–N–C at 2.66 Å (uncorrected), correlated with a k-value of ∼7.0 Å^–1^ (Fig. 2j), and that at 2.75 Å (uncorrected), correlated with a k-value of ∼7.9 Å^–1^ (Fig. 2l), could be attributed to Cu–Fe bonding. Theoretical spectra of the CF-HNCS-1 and CF-HNCS-2 structural models are in good agreement with the experimental data (Fig. S6), which further implies the formation of Fe–Cu bonds. Although the second intensity maximum could also be speculated to be due to Cu–Cu or Fe–Fe bonding, such a possibility can be ignored due to the substantially higher catalytic activity of the heteronuclear Cu–Fe dual-atom catalyst presented in previous reports, and the formation of an ideal structure in natural human cytochrome c oxidase (PDB 5Z62) [25,32]. The WT analysis demonstrates that Cu–Fe–N–C truly contains dual atomic sites, consistent with the EXAFS fitting parameters. These structural characterizations show that CF-HNCS has Cu–Fe active sites similar to those of HCOs.

### Catalytic performance

**Figure 3.**
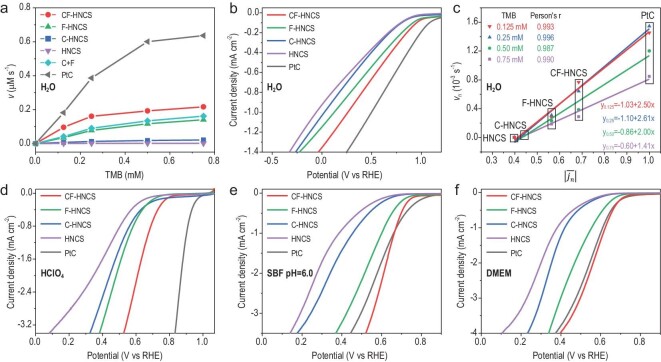
Catalytic performance of CF-HNCS. (a) Velocity (*v*) of the chromogenic reaction in the presence of various samples in deionized water. (b) LSV curves of various samples in O_2_-saturated deionized water. (c) Linear fitting between the non-dimensionalized velocity (*v*_n_) of the chromogenic reaction at various TMB concentrations in deionized water and the absolute value of the normalized mean current density from 1 to 0 V in the ORR in O_2_-saturated deionized water. (d–f) LSV curves of CF-HNCS and reference samples in (d) O_2_-saturated 0.1 M HClO_4_ solution, (e) O_2_-saturated SBF solution and (f) O_2_-saturated DMEM solution.

The synergistic catalytic effect between copper and iron atoms in the CF-HNCS catalysts may endow the catalysts with excellent oxidase-like performance. To investigate the oxidase-like activities of the model catalysts, we employed the oxidation of 3,3^′^,5,5^′^-tetramethylbenzidine (TMB) as a model chromogenic reaction. In deionized water, the velocities of the chromogenic reaction (Fig. 3a) show the oxidase-like activity order of CF-HNCS > C-HNCS + F-HNCS > F-HNCS > C-HNCS ≫ HNCS, indicating that the synergistic effect and the active metal atom are two important factors for catalytic activity.

In fuel cells, the cathode receives oxidants (often oxygen), and the anode receives reductants, such as some organic molecules [33,34]. The ORR is the half reaction in the oxidization of the reductant by O_2_. From a chemical reaction viewpoint, if the ORR activity of the catalyst is elevated, then its capacity for catalysing organic oxidation by O_2_ would be enhanced under the same reaction conditions, especially when the rate-determining steps (RDSs) are in the O_2_ desorption and adsorption processes. Considering the remarkable ORR activity of commercial Pt/C, Pt/C may exhibit excellent activity in catalysing O_2_ reduction to oxidize organic molecules. The velocity of the chromogenic reaction in deionized water (Fig. 3a) and the time-dependent absorbance of TMB in NaAc buffer solution (Fig. S8a) show that the oxidase-like activity of commercial Pt/C is significantly higher than that of CF-HNCS. Nevertheless, notably, the calculated turnover frequency of Cu–Fe in CF-HNCS is ∼0.93 times that of Pt in Pt/C, revealing the outstanding oxidase-like activity of CF-HNCS. Then, we investigated the ORR activity of all samples by steady-state linear sweep voltammetry (LSV) in various solutions at room temperature (∼25°C). The obtained order of ORR activity is Pt/C > CF-HNCS > F-HNCS > C-HNCS ≫ HNCS in both O_2_-saturated deionized water and O_2_-saturated NaAc buffer solution (Fig. 3b and Fig. S8b), which is in excellent agreement with the oxidase-like activity order identified by TMB (Fig. 3a and Fig. S8c), suggesting that the oxidase-like and ORR activities may follow the same mechanism and have the same active sites. The non-dimensionalized velocity (*v*_n_) of the chromogenic reaction at various TMB concentrations and the absolute value of the normalized mean current density ($| {\overline {{j_{\rm{n}}}} } |$) from 1 to 0 V in the ORR are used to represent the oxidase-like and ORR activities of various catalysts, respectively. Figure 3b and f show the excellent fitting linearity between *v*_n_ and $| {\overline {{j_{\rm{n}}}} } |$ in both deionized water and NaAc buffer solution, in which all Pearson correlation coefficients (Pearson's r) are >0.985. The slopes and intercepts increase with increasing TMB concentration from 0.75 to 0.25 mM, indicating that the enzyme becomes saturated when the substrate concentration increases. In particular, the linear relations at 0.25 and 0.125 mM are almost the same, demonstrating that y_0.125_ and y_0.25_ could well describe the relation between the oxidase-like and ORR activities in the linear part of enzyme reactions. Thus, $| {\overline {{j_{\rm{n}}}} } |$ is an effective descriptor for the catalytic activity of oxidase mimics. The colorimetric reaction of *o*-phenylenediamine (OPD) also shows a positive correlation with $| {\overline {{j_{\rm{n}}}} } |$, proving the generality of the descriptor (Fig. S9). Based on the above analysis, we propose that the oxygen reduction electrocatalysts possess homologous oxidase-like activity by sharing the same activity origins, so the conventional LSV technique is readily applicable to describing oxidase-like activity, especially when the RDS of oxidase-like activity is in the oxygen activation process and the catalysts used for comparison are of the same kind or at least have similar conductivities. Furthermore, the ORR activities also follow the order of Pt/C > CF-HNCS > F-HNCS > C-HNCS ≫ HNCS in an O_2_-saturated 0.1 M HClO_4_ solution (Fig. 3d), implying that a large number of reported oxygen reduction electrocatalysts with remarkable activities might be promising candidates for oxidase mimics.

The currently popular electron spin resonance (ESR), fluorescence and chromogenic methodologies for assessing oxidase-like activity are difficult to apply in high-glucose Dulbecco's modified Eagle medium (DMEM), which is used in cell culture but complicated in composition. However, the LSV technique works well in DMEM. The ORR activity order in O_2_-saturated simulated body fluid (SBF) and O_2_-saturated DMEM solutions is CF-HNCS > Pt/C > F-HNCS > C-HNCS ≫ HNCS (Fig. 3e and f). Interestingly, the ORR activity of CF-HNCS here is higher than that of Pt/C in DMEM and SBF solutions, indicating that glucose and anions, such as chloride and phosphate anions, may poison Pt/C [35,36] and that CF-HNCS could be a better cancer therapeutic catalyst owing to the great anti-poisoning effect and high-performance catalytic activity. To compare the stabilities of CF-HNCS and Pt/C, a long-term cycling test between 0.1 and 1.0 V (vs. RHE) was performed in SBF and DMEM. Figure S11 shows that the decrease in the ORR electrocatalytic activity for CF-HNCS is much slower than that for Pt/C, indicating the much better anti-poisoning effect of CF-HNCS than Pt/C. Cyclic voltammograms (CVs) at the CF-HNCS electrodes were obtained to reveal the influence of solutions on electrochemical performance. As shown in Fig. S12a, the numbers of oxidation and reduction peaks differ in different solutions due to the changed solute, revealing that the DMEM solution offers the maximum number of redox couples and that these redox reactions can be catalysed by CF-HNCS. Based on this, the CV technique could be well used to identify redox couples catalysed by oxidase mimics in different solutions.

From an electrochemical viewpoint, reactive oxygen species (ROS) are generated as intermediates during the ORR [37], which is consistent with the pathway of oxidase-based enzymatic catalysis [14,38–40]. Therefore, we employed ESR to monitor possible active intermediate production. The six-band spectra originate from 5-tert-butoxycarbonyl-5-methyl-1-pyrroline-N-oxide (BMPO)/•OOH, and the band intensities follow the order of CF-HNCS > F-HNCS > C-HNCS ≫ HNCS (Fig. S12b), demonstrating that the ROS generation performance of these model SACs corresponds well to the ORR catalytic activity characterized by LSV and the oxidase-like activity characterized by TMB. In addition, significant dihydroethidium (DHE) fluorescence enhancement was observed in the presence of CF-HNCS, demonstrating the generation of O_2_•^−^ (Fig. S12c). Glutathione (GSH) is a reducing species in tumour cell redox homeostasis [41], and the fluorescence intensity reaches its maximum when adding both CF-HNCS and GSH, suggesting that GSH can act as a promoter in the oxidation of DHE. This result also implies that GSH can be oxidized and may promote CF-HNCS catalysis towards the oxidation of biomolecules in cells.

### DFT calculations

**Figure 4.**
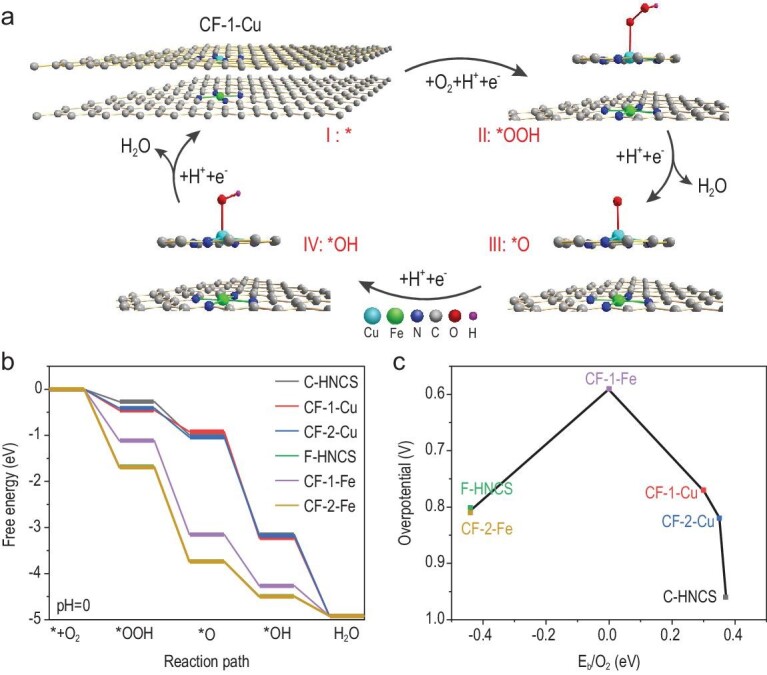
DFT calculations of the catalytic activity. (a) Proposed reaction pathways of the ORR on CF-HNCS-1 at CF-1-Cu. The grey, dark blue, light blue, green, red and black balls represent the C, N, Cu, Fe, O and H atoms, respectively. (b) Free energy profiles of the ORR at different active sites under common conditions (U = 0, T = 298 K and P = 1 bar). (c) Thermodynamic relation of the ORR overpotentials to the O_2_ binding energies at different active sites.

DFT calculations were performed to investigate the mechanism and shared origins of the oxidase-like/ORR activities. We hypothesize a 4e^−^ reduction pathway in which an O_2_ molecule is first adsorbed and protonated to form ^*^OOH on top of central Cu or Fe atoms. Then, ^*^OOH associates with H^+^ and dissociates into ^*^O and H_2_O. Next, ^*^O is protonated to form ^*^OH. Finally, ^*^OH associates with H^+^ to generate the final product H_2_O (Fig. 4a and Fig. S13).

The free energy profiles in Fig. 4b show that the ORR overpotentials for C-HNCS, CF-HNCS-1-Cu (CF-1-Cu), CF-HNCS-2-Cu (CF-2-Cu), F-HNCS, CF-HNCS-1-Fe (CF-1-Fe) and CF-HNCS-2-Fe (CF-2-Fe) were estimated to be 0.96 V, 0.77 V, 0.82 V, 0.80 V, 0.59 V and 0.81 V, respectively. Based on the free energy values, the calculated ORR activity order is CF-HNCS-1 > CF-HNCS-2 > F-HNCS > C-HNCS, in accordance with the experimental findings. Figure 4b shows that the first step is the RDS on Cu, while the fourth step is the RDS on Fe. Therefore, the superior ORR activity of dual-atom CF-HNCS-1 compared to the other SACs is ascribed to the enhanced adsorption of ^*^OOH at the Cu site, which facilitates the protonation of O_2_ on CF-1-Cu, and to the weakened adsorption of ^*^OH at the Fe site, which facilitates the removal of H_2_O from CF-1-Fe. In CF-HNCS-2, the overpotential of CF-2-Fe slightly increases compared to the Fe site in F-HNCS, while that of CF-2-Cu significantly decreases to 0.77 V, which is even lower than that of the Fe sites in F-HNCS and CF-HNCS-2. Therefore, the dual Cu and Fe atomic system with the lowest overpotential demonstrates the highest ORR activity compared to the corresponding single-atom Cu or Fe systems.

Figure 4c shows that the thermodynamic relation between the ORR overpotentials and the O_2_ binding energies roughly forms a volcano-type curve. On the left side, the fourth step (^*^OH + H^+^ + e^–^ → H_2_O) is the RDS, suggesting that the excessively high O_2_ binding energy makes the adsorption and dissociation of ^*^OH species less favourable. In contrast, the first step (O_2_ + H^+^ + e^–^ → ^*^OOH) is the RDS on the right side, revealing that the overly weak adsorption and activation of O_2_ molecules increase the difficulty of the protonation of O_2_. CF-1-Fe has an appropriate O_2_ binding energy, endowing the catalyst with the optimal ORR activity. These results imply that optimizing the binding strength of O_2_ on catalytic sites is an efficient way to achieve excellent ORR activity. Finally, the free energy profiles of the ORR under different pH values (1.0, 4.5, 6.0, 7.4) are given in Fig. S14. The most energetically favourable pH value is 1.0, implying that a reasonably strong acidic environment is beneficial for improving catalytic performance. Figure S14d shows that the reaction process has a negative Gibbs free energy (−3.17 eV) and a surmountable apparent energy barrier (0.17 eV), even at pH = 7.4. Accordingly, the DFT results indicate that the ORR being effectively catalysed at active sites is both thermodynamically feasible and kinetically favourable from pH = 0 to 7.4.

We have probed the catalytic mechanism and the shared origins of the oxidase-like/ORR activities of the model SACs at the atomic level and identified that the metal active sites and the Cu–Fe synergistic effect are two key factors for catalytic activity. In addition, CF-HNCS-1 has the most favourable catalytic structure, indicating that biomimetic synthesis is an effective way to develop next-generation oxidase-like/ORR catalysts.

### *In vitro* catalytic therapeutic performance

**Figure 5.**
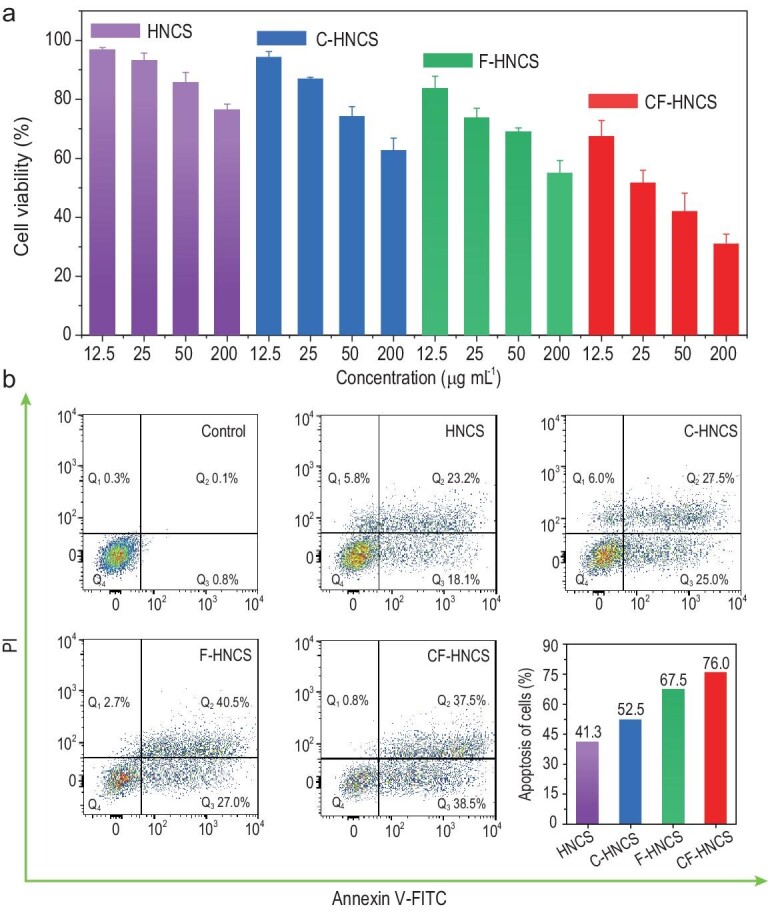
*In vitro* assessment of catalytic therapeutic efficacy. (a) Viability of 4T1 cells after incubation with different concentrations of materials. (b) Annexin V-FITC/PI staining analysis of 4T1 cells incubated with different materials (200 ppm).

The intracellular ROS generation capacity of CF-HNCS was detected by flow cytometry with DHE. The 4T1 cells treated with CF-HNCS exhibit the strongest DHE fluorescence intensity (Fig. S15), indicating that the catalysts are capable of catalysing significant production in intracellular ROS. Encouraged by the excellent oxidase-like activity of CF-HNCS catalysts for the generation of ROS and the oxidation of organic molecules such as GSH, we further explored the *in vitro* anticancer efficacy. First, the Cell Counting Kit-8 (CCK-8) assay was used to investigate catalytic therapeutic efficacy against 4T1 tumour cells. As expected, a dose-dependent cellular killing effect was observed, as shown in Fig. 5a. Moreover, the 4T1 tumour cell viability reduction rates reached 58% and 69% at CF-HNCS concentrations of 50 and 200 ppm, respectively, in which the metallic Cu and Fe concentrations were as low as 0.93 and 3.72 ppm, respectively. More interestingly, the cellular killing effects also follow the order of CF-HNCS > F-HNCS > C-HNCS > HNCS, implying that the oxidase-like activities of the catalysts are responsible for the significant anticancer effects. Due to the relatively low redox potential of O_2_•^–^ and the complicated cell environment, we were not able to directly detect the oxidase-like activities of the catalysts in the cell. Flow cytometric analysis was further conducted to study the apoptosis of 4T1 cancer cells by staining with annexin V-FITC and PI. Necrotic (Q_1_), late apoptotic (Q_2_), early apoptotic (Q_3_) and live (Q_4_) cells are presented in the four quadrants in Fig. 5b. A similar trend is also obtained for the cell apoptosis order. The sum of early and late cellular apoptotic rates is up to 76.0% in the CF-HNCS group, showing prominent therapeutic efficacy *in vitro*.

### *In vivo* catalytic therapeutic performance

**Figure 6.**
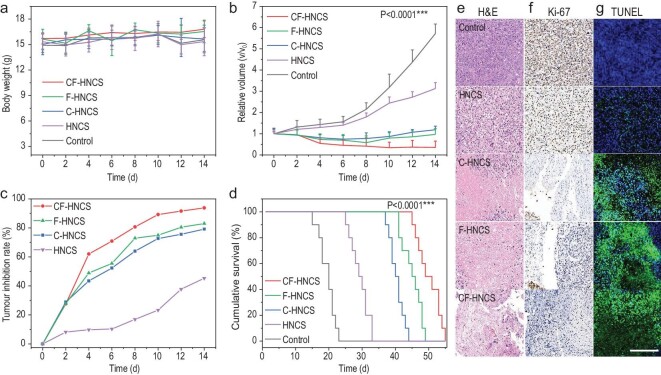
*In vivo* assessment of catalytic therapeutic efficacy. (a) Body weight changes, (b) tumour volume changes, (c) RTI rates and (d) survival curves of 4T1 xenografted tumour-bearing mice (*n* = 10 per group) treated with different catalysts. Data were assessed by Student's two-sided t-test. ^*^P < 0.1, ^**^P < 0.05, ^***^P < 0.005 compared to the control group. (e) Representative H&E staining sections for nuclear dissociation, (f) Ki-67 immunohistochemical staining sections for cellular proliferation and (g) TUNEL staining sections for necrosis, of tumour tissue collected 14 days after treatments. Scale bar: 400 μm.

Compared to conventional injection methods, the microneedle (MN) patch offers a painless and minimally invasive self-administration route, avoiding the risk of bleeding and injury [42,43]. In this study, we fabricated a polyvinyl pyrrolidone (PVP)-based MN patch for delivery of CF-HNCS catalysts (Fig. S16a–e). First, different doses of CF-HNCS catalysts were confirmed to have excellent biocompatibility in healthy Kunming mice (Fig. S18a–c). A more detailed analysis of the biocompatibility experiment is given in the above discussion. Furthermore, the therapeutic efficacies of these synthesized catalysts for catalytic tumour therapy were assessed in 4T1 xenografted tumour-bearing mice. CF-HNCS-MN was intratumourally injected, and then the MN dissociated and released catalysts for cancer therapy. There were slight fluctuations in the weights of these tumour-bearing mice, demonstrating the good biocompatibility of the MN (Fig. 6a). Importantly, the CF-HNCS, F-HNCS and C-HNCS catalysts show remarkable effects in suppressing tumour growth (Fig. 6b). The relative tumour inhibition (RTI) rates compared to the control group were calculated to quantify the therapeutic performance. The RTI rates of the experimental groups treated with catalysts gradually increase and reach their maxima at the end of the observations, indicating the effective long-term suppression of tumour growth by the catalysts (Fig. 6c). In particular, the RTI rate reaches 94% on day 14 in the CF-HNCS group. In addition, the overall survival length in the CF-HNCS group is more than twice that in the control group, showing a significant elevation in survival rates after catalyst treatment (Fig. 6d). The therapeutic performance was also evaluated by histological analysis using pathological haematoxylin and eosin (H&E) (Fig. 6e), Ki-67 antibody (Fig. 6f) and terminal deoxynucleotidyl transferase dUTP nick end labelling (TUNEL) staining (Fig. 6g). After catalytic therapy, the tumour tissues exhibit prominent cell fibrosis, apoptosis and necrosis (Fig. 6e), significantly suppressed proliferation (Fig. 6f), serious cell apoptosis and fragmentation of DNA (Fig. 6g). Most importantly, the catalytic therapeutic outcomes *in vivo* also follow the order of CF-HNCS > F-HNCS > C-HNCS > HNCS, confirming that the oxidase-like/ORR activities of materials are responsible for the inhibition of tumours. These results demonstrate that we could search for and screen effective therapeutic nanocatalysts by assessing their oxidase-like, i.e. ORR, activity before *in vivo* experiments, which is expected to accelerate the screening of potential therapeutic nanocatalysts.

## CONCLUSION

**Scheme 1.**
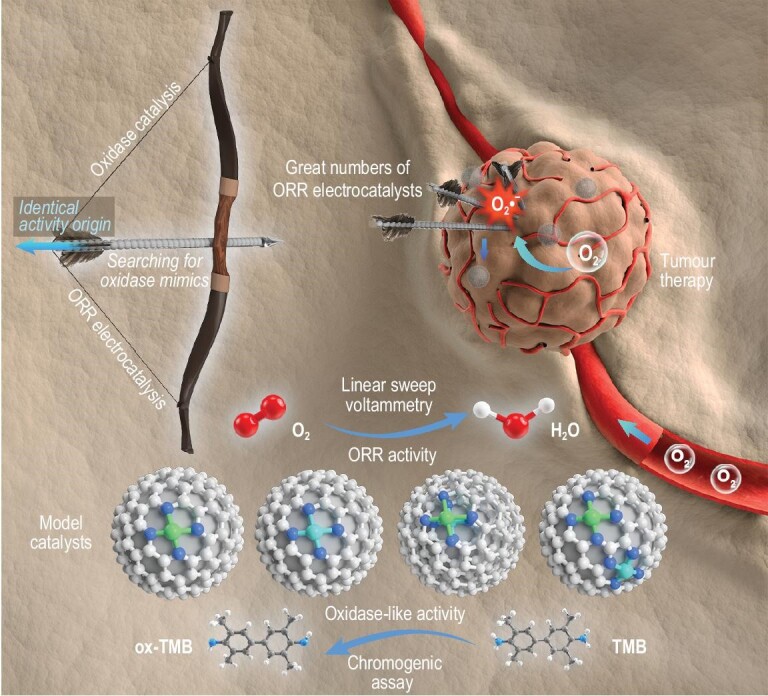
Schematic diagram of the bridging between oxidase catalysis and ORR electrocatalysis. Based on the identical activity origin and model single-atom catalysts, a facile electrochemical methodology (linear sweep voltammetry) has been developed and a normalized mean current density has been proposed for rapid oxidase-like catalyst evaluation. As a result, great numbers of high-activity ORR electrocatalysts are expected to be promising candidates for oxidase mimics. These findings are inspiring and useful in the search for oxidase mimics for tumour therapy, and encourage us to clarify the relationship between enzyme- and electrocatalysis in the future.

In summary, we present a proof-of-concept study bridging biological oxidase-like and electrochemical ORR catalysis, both experimentally and theoretically, by using model SACs (Scheme 1). Importantly, the oxidase-like and electrochemical ORR catalytic activities have been demonstrated to be linearly correlated with each other based on their shared identical activity origins. Therefore, electrocatalytic characterizations, such as LSV technique, could be reliable, facile and efficient techniques to search for oxidase mimics, especially when the RDS of oxidase-like activity is in the oxygen activation process and the catalysts used for comparison are of the same kind or at least have similar conductivities. Quantitatively, the normalized mean current density $| {\overline {{j_{\rm{n}}}} } |$ of the cathodic ORR has been found to be an apparent descriptor for characterizing the oxidase-like activity of the catalyst. As a result, great numbers of high-activity oxygen reduction electrocatalysts are expected to possess prominent oxidase-like activity, which encourages us to find more types of electrochemical catalysts and verify their enzyme-like catalytic activity in the future. Meanwhile, the regulation strategies for enhancing the electrocatalytic ORR activity of metal-N-C SACs, such as modulating the central metal atoms, the coordinated atoms and the environmental atoms, are expected to have great potential for boosting the oxidase-like activity of catalysts. Furthermore, both experimental and theoretical calculations demonstrate that the biomimetic heterobinuclear Cu–Fe centres are well balanced, i.e. in terms of the optimal adsorption strength of oxygenated species on the active sites and the significant synergistic effect between the two kinds of metal atoms, which is responsible for the outstanding catalytic activity. Our findings highlight the prospect of designing high-performance biomimetic enzymes by facilely designing and synthesizing desirable metal centres as active sites for the ORR, and pave the way for biomedical and environmental applications of electrocatalysts, such as in tumour therapy, antibiosis and oxidation degradation of organic pollutants.

## Supplementary Material

nwac022_Supplemental_File
