# Supplementary material for: Bridging oxidase catalysis and oxygen reduction electrocatalysis by model single-atom catalysts
Source: Natl Sci Rev. 2022 Feb 23;9(10):nwac022. doi: 10.1093/nsr/nwac022 (PMC9671664; doi:10.1093/nsr/nwac022)
Supplement: nwac022_Supplemental_File [file nwac022_supplemental_file.docx]

Supplementary Information for

**Bridging** **Oxidase Catalysis and** **Oxygen Reduction Electrocatalysis** **by Model Single-Atom Catalysts**

Xiangyu Lu (逯向雨)^1,2,3^, Shanshan Gao (高珊珊)^4^, Han Lin (林翰)^1,3^_,_ Han Tian (田汉)^1,2^, Deliang Xu (徐德良)^1^, and Jianlin Shi (施剑林)^1,2,3*^

^1^State Key Lab of High Performance Ceramics and Superfine Microstructure, Shanghai Institute of Ceramics, Chinese Academy of Sciences, Shanghai 200050, P. R. China.

^2^Center of Materials Science and Optoelectronics Engineering, University of Chinese Academy of Sciences, Beijing 100049, P. R. China.

^3^Shanghai Tenth People's Hospital, Shanghai Frontiers Science Center of Nanocatalytic Medicine，The Institute for Biomedical Engineering and Nano Science，School of Medicine, Tongji University, Shanghai 200092, China.

^4^ School of Public Health, Shanghai Jiao Tong University School of Medicine, Shanghai 200025, P. R. China.

E-mail: jlshi@mail.sic.ac.cn

***The supplementary information includes:***

***Part A.*** Supplementary methods

***Part B.*** Supplementary scheme

***Part C.*** Supplementary figures

***Part D.*** Supplementary tables

***Part E.*** Supplementary discussions

***Part F.*** Supplementary references

**Part A. Supplementary methods**

**Methods**

**Chemicals.** Copper(II) acetate monohydrate (Cu(OAc)_2_·H_2_O), α,α'-dibromo-p-xylene (97%), Polyvinylpyrrolidone powder (PVP, average relative molecular mass of 30,000 (PVP30)), 3,3′,5,5′-tetramethylbenzidine dihydrochloride hydrate (TMB), o-phenylenediamine (OPD), dihydroethidium (DHE) were purchased from Shanghai Sigma-Aldrich Trading Co. Ltd. Iron(II) acetate tetrahydrate (Fe(OAc)_2_·4H_2_O) was purchased from Shanghai Macklin Biochemical Co. Ltd. Ammonium hydrogen difluoride (NH_4_HF_2_), NH_3_·H_2_O (28 wt%), tetraethyl orthosilicate (TEOS), 5,10,15,20-tetra(4-(imidazol-1-yl)phenyl)porphyrindine (TIPP), and hydrochloric acid (HCl, 37 wt%) were purchased from Shanghai Chemical Reagents. Reduced GSH, hydrogen peroxide aqueous solution (H_2_O_2_, 30 wt%) and dimethyl sulfoxide (DMSO) were obtained from Aladdin. Acetic acid and sodium acetate trihydrate were obtained from Alfa Aesar. Carbon black (XC-72) and commercial 20 wt% Pt/C were purchased from Shanghai HEPHAS Energy Equipment Co. Ltd. A Nafion D-520 dispersion (5 wt%) was purchased from DuPont China Holding Co. Ltd. Phosphate-buffered saline (PBS) was purchased from Shanghai Double-helix Biotech Co. Ltd. Simulated body fluid (SBF) was purchased from Dongguan Xinheng Co. Ltd. Dulbecco’s Modified Eagle Medium (DMEM) was purchased from GE Healthcare Life Sciences. The Annexin V FITC/PI Apoptosis Detection Kit and BMPO were purchased from Shanghai Dojindo Co. Ltd. All chemicals were used directly without further purification.

**Characterization.** HAADF-STEM, HR-TEM, and corresponding EDS mappings were carried out on a JEOL ARM-300F instrument at 300 kV. TEM images were measured on a JEM-2100F transmission electron microscope. SEM images were obtained using FE-SEM (Hitachi SU8240). XRD patterns were acquired using a Rigaku D/MAX-2550 V X-ray powder diﬀractometer with a Cu Kα line (λ = 1.5418 Å). Raman spectra were measured on a DXR Raman microscope (Thermal Scientific). N 1s XPS spectra were recorded on an ESCAlab250 (Thermal Fischer). FT-IR spectra were acquired on a Nicolet 7000-C spectrometer. The concentrations of Cu and Fe were analysed by inductively coupled plasma optical emission spectrometry (ICP-OES) (Agilent 700 Series, Agilent Technologies). ESR experiments were performed on a JEOL-FA200 ESR spectrophotometer. Fluorescence measurements were carried out on a Hitachi F-4600 fluorescence spectrophotometer. Chromogenic reaction studies were conducted on a Microplate Reader SpectraMax M2 (Molecular Devices). Flow cytometry analysis was performed on a BD LSRFortessa cytometer.

**Preparation of the SiO_2_ template.** Briefly, 20 ml of deionized water, 5 ml of NH_3_·H_2_O (28 wt%), and 60 ml of absolute ethanol were premixed and magnetically stirred (~ 550 rpm) at 30 °C for 30 min, followed by the dropwise addition of 5 ml of TEOS. After a 10 h hydrolysis/condensation reaction, ≈ 300 nm silica nanoparticles were generated by centrifugation and washed with ethanol several times.

**Preparation of Cu-TIPP and Fe-TIPP.** In a typical procedure ^[1]^, 119.8 mg of Cu(OAc)_2_·H_2_O and 50 ml of N,N-dimethylformamide (DMF) were mixed to form a clear solution, which was quickly added to 50 ml of DMF including 0.175 g of TIPP in a 250 ml round bottom flask at approximately 25 °C. The above mixture was refluxed for 4 h at 175 °C, followed by the evaporation of 80 ml of DMF. The residual solution was cooled to approximately 25 °C, and 200 ml of freezing water was added. After 1 h, the precipitates in the above mixture were centrifuged and washed three times using water. Finally, Cu-TIPP was dried in a vacuum drying chamber at 120 °C for 4 h.

The same procedure was repeated to synthesize Fe-TIPP but using 0.60 mmol Fe(OAc)_2_·4H_2_O instead of Cu(OAc)_2_·H_2_O.

**Preparation of the CF-HNCS nanocatalyst.** SiO_2_@Cu/Fe-TIPP/TIPP-polymer was synthesized *via* the modified method reported by Wang et al ^[1, 2]^. First, 500 mg of SiO_2_, 15 mg of Cu-TIPP, and 15 mg of Fe-TIPP were mixed with 100 ml of DMF at approximately 25 °C in a round bottom flask, followed by heating to 175 °C and stirring for another 1 h. Then, 270 mg of TIPP was added into the above solution and stirred at 110 °C for another 1 h. Next, 20 ml of DMF including 179.5 mg of *α*,*α*’-dibromo-p-xylene was quickly added into the mixture under rapid stirring (~ 800 rpm) at approximately 25 °C. The obtained mixture was stirred at 110 °C for 24 h to form SiO_2_@Cu/Fe-TIPP/TIPP-polymer. The product was centrifuged and washed with DMF and ethanol several times and then dried for 12 h in a vacuum drying chamber at 80 °C. Finally, the SiO_2_@Cu/Fe-TIPP/TIPP-polymer was pyrolyzed at 800 °C under a H_2_/Ar (a= 5/95) atmosphere for 3 h with a ramping rate of 5 °C min^–1^. After natural cooling to room temperature, the obtained product was etched by a 6 M NH_4_HF_2_ aqueous solution for two days at 60 °C to remove the SiO_2_ core. The obtained CF-HNCS catalyst was centrifuged and washed with water several times. HNCS, C-HNCS, and F-HNCS were similarly synthesized by using 300 mg of TIPP, 15 mg of Cu-TIPP + 285 mg of TIPP, and 15 mg of Fe-TIPP + 285 mg of TIPP, respectively, in the reaction system.

**XAFS measurements and analysis.** XAFS spectra of the Cu K-edge and Fe K-edge were acquired in fluorescent mode using a Lytle detector at beamline BL14W1 in the Shanghai Synchrotron Radiation Facility (SSRF) ^[3]^. The storage rings of SSRF were operated with a current between 150 and 210 mA at an energy of 3.5 GeV and monochromatized using a Si (111) double crystal monochromator. The monochromator energy was calibrated using a Cu foil or Fe foil.

The XAFS raw data were background-subtracted and normalized in Athena (version 0.9.25) ^[4]^. Then, Fourier transform analysis was performed in Artemis (version 0.9.25). An R range of 1–3 Å, k^3^ weighting, and a k-range of 3–12 Å^–1^ were employed to fit the Cu K-edge EXAFS spectra. An R range of 1–3 Å, k^2^ weighting, and a k-range of 2–10 Å^–1^ were employed to fit the Fe K-edge EXAFS spectra. The amplitude reduction factor (S_0_^2^) was confirmed by fitting the experimental Cu foil or Fe foil data. Parameters such as the coordination number (CN), bond length (R), Debye-Waller factor (*σ*^2^), and E_0_ shift (ΔE_0_) were allowed to change freely during the fitting process.

For WT analysis, the χ(k) obtained from Athena was imported into the Hama Fortran code ^[5]^. The parameters were selected as follows: k weight, 2; k range, 0–11 Å^–1^; R range, 1–4 Å. The Morlet function with κ = 10 and *σ* = 1 was applied as the mother wavelet to provide the overall distribution.

Simulations of the XANES were performed using the finite difference method as implemented within the Finite Difference Method Near Edge Scattering (FDMNES) package using a free form SCF potential of radius 6.0 Å around the absorbing atom. Broadening contributions due to the finite mean-free path of the photoelectron and to the core-hole lifetime were accounted for using an arctangent convolution.

**Oxidase-like activity of CF-HNCS.** The oxidation of TMB with an absorbance peak at 652 nm was recorded by a UV–vis spectrophotometer to study the oxidase-like activity of the catalysts. For the kinetic measurements, a certain amount of CF-HNCS (ﬁnal concentration: 10 μg ml^–1^) and different amounts of TMB (ﬁnal concentration: 0, 0.125, 0.25, 0.50, 0.75, 1, 1.5, 5, 10, 25 mM) were mixed in 1.0 ml of deionized water. Effects of temperature on the catalytic activity was carried in deionized water (TMB, 0.50 mM). In HAc-NaAc buffer solution (0.1 M, pH 4.5), the tests were performed with CF-HNCS (ﬁnal concentration: 10 μg ml^–1^) and TMB (ﬁnal concentration: 0.1 mM). Effects of pH on the catalytic activity was carried in HAc-NaAc buffer solution (0.1 M, pH 3.0, 4, 4.5, 5.0, 6.0, 7.0, 9.0, 12.0) with TMB (0.50 mM). The derivatives of the absorbance curve were calculated as the velocities. For the OPD colorimetric reaction, a certain amount of catalysts (ﬁnal concentration: 250 μg ml^–1^) and different amounts of OPD (ﬁnal concentration: 200 mM) were mixed in 1.5 ml of deionized water. After the mixture incubated for 60 min, UV-vis absorption spectra were acquired. The catalytic activities of HNCS, C-HNCS, F-HNCS, and Pt/C were also investigated for comparison. For the detection of superoxide radicals, the spin trap BMPO (ﬁnal concentration: 25 mM) and CF-HNCS (ﬁnal concentration: 20 μg ml^–1^) were added to 0.2 ml of HAc-NaAc buffer solution (0.1 M, pH 4.5). After the mixture was aggressively vortexed for 30 s and incubated for 8 min, ESR spectra were acquired. The oxidation of DHE with a fluorescence peak at 625 nm under excitation at 520 nm was also recorded by ﬂuorescence spectrometry to study the oxidase-like activity of the catalysts. In this test, CF-HNCS (ﬁnal concentration: 20 μg ml^–1^) and DHE (ﬁnal concentration: 100 μM) were added to 3 ml of deionized water with or without GSH (ﬁnal concentration: 300 μM).

**Electrochemical measurements.** Cyclic voltammetry (CV) and linear sweep voltammetry (LSV) tests were conducted on a CHI 760E electrochemical workstation (Shanghai Chenhua Instrument Factory, China), in which the Ag/AgCl electrode (in 3 M KCl solution) and carbon rod were regarded as the reference electrode and counter electrode, respectively. Deionized water, HAc-NaAc buffer solution (0.1 M, pH 4.5), SBF (pH 7.4), DMEM (pH 7.4) or 0.1 M HClO_4_ aqueous solution was used as the electrolyte. A rotating disk electrode (glassy carbon electrode) with an area of 0.196 mm^2^ served as the substrate for the working electrodes. All potentials in this article were calibrated relative to the reversible hydrogen electrode (RHE) according to the Nernst equation (E (versus RHE) = E (versus Ag/AgCl) + 0.209 + 0.059 pH).

For the ORR experiments, the catalyst ink was developed by ultrasonically dispersing 10.0 mg of catalysts and 10 μl of Nafion (5 wt%) into 900 μl of isopropanol and 90 μl of deionized water. When carrying out the measurements, 10 μl of catalyst ink was dropped on the glassy carbon electrode and naturally dried to form a thin-film electrode. The electrolytes were saturated with high-purity oxygen by bubbling O_2_ for at least 30 min before the ORR experiments. CV tests were performed to activate the catalysts at a scan rate of 100 mV s^−1^ at 25 °C. Then, LSV tests were performed at a scan rate of 5 mV s^−1^ under a rotation rate of 1600 rpm.

The formulas *v*_n_ = *v*/*c* and $\left| \overline{j_{n}} \right|=\left| \overline{j} \right|/\left| \overline{j_{\mathrm{PtC}}} \right|$were employed to calculate the nondimensionalized velocity (*v*_n_) of the chromogenic reaction and the absolute value of the normalized mean current density ($\left| \overline{j} \right|$), respectively, where *c* and *j* represent the concentration of TMB and the mean current density over a range of voltages.

**Calculation details.** The Vienna ab initio simulation package (VASP) program^[6, 7]^ and Perdew, Burke and Ernzerhof (PBE) functional^[8]^ were applied for calculations. The kinetic cutoff energy level was set as 500 eV, while the gamma point was regarded as the k-point. Accounting for the dispersion interaction, Grimme’s D3 correction was used^[9]^. Models of C-HNCS, F-HNCS, and CF-HNCS-2 with a 9×9×1 supercell were chosen for energy calculations, while the model of CF-HNCS-1 used a 9×9×2 supercell. The general four-electron ORR reaction under acidic conditions was split into the following elementary steps:

The asterisk (*) represents the active site of the catalyst.

**Cell culture.** Murine breast cancer 4T1 cells were purchased from the Cell Bank of Shanghai Institutes for Biological Sciences, Chinese Academy of Sciences. The 4T1 cells were cultured in high-glucose DMEM containing 10% foetal bovine serum (FBS) and 1% streptomycin/penicillin in a humidified incubator at 37 °C under 5% CO_2_.

**Cellular ROS assay**

To monitor the intracellular production of ROS, 4T1 cells were incubated with fresh medium containing CF-HNCS, F-HNCS, C-HNCS, or HNCS for 6 h in 6-well plates. Then, the harvested cells were incubated with 5 μM dihydroethidium for 30 min at room temperature, and the ﬂuorescence was measured by ﬂow cytometry with λ_ex_ = 488 nm and λ_em_ = 610 nm.

***In vitro* cell viability assay.** A standard CCK-8 viability kit assay (Dojindo Laboratories, Shanghai) was conducted to evaluate the *in vitro* catalytic therapeutic performance of Cu-HNCS. One hundred microlitres of 4T1 cell solution was seeded in a 96-well microplate at a density of 5000 cells/well for 12 h. To mimic the acidic microenvironment in a solid tumour, HCl was employed to regulate the pH value of DMEM from 7.4 to 6.0. Then, different concentrations (0, 12.5, 25, 50, 200 μg ml^−1^) of Cu-HNCS were added to the 96-well microplates. The cells were cultured for another two days. Finally, the standard CCK-8 assay procedure was performed to assess the cell viability (n = 6) at 450 nm by a microplate reader.

***In vitro* flow cytometric apoptosis assay.** One thousand microlitres of 4T1 cell solution was seeded in a 6-well plate at a density of 1×10^5^ cells/well for 12 h to adhere. After coincubation with 1000 μl of acidic DMEM containing CF-HNCS (100 μg ml^−1^) for another two days, the 4T1 cells were gathered by trypsin and stained with Annexin V-FITC and PI according to the protocol in a test tube. Finally, the fluorescence intensity was monitored for flow cytometric analysis.

***In vivo* toxicity assay.** The animal experiments followed procedures approved by the Animal Care Ethics Commission of Shanghai Tenth People’s Hospital, Tongji University School of Medicine. Twenty female Kunming mice (~23 g) obtained from Shanghai Laboratory Animal Center (SLAC) were randomly divided into four groups: (1) PBS (control group), (2) CF-HNCS in PBS (5 mg/kg), (3) CF-HNCS in PBS (10 mg/kg), and (4) CF-HNCS in PBS (20 mg/kg). Different doses of CF-HNCS were then subcutaneously administered to the mice in the corresponding group. Their body weights were measured every three days for a month. Systematic serum biochemical assays and blood panel analysis were performed at the end of the assessment. The major organs, including the heart, liver, spleen, lung, and kidney, were collected at day 30, ﬁxed in 10% formalin, and then stained with H&E.

**Preparation of MN patches.** Typically, 0.8 g of PVP and 0.2 g of CF-HNCS were added to deionized water (0.5 ml) to form a uniform mixture. Then, 50 μl of this mixture was deposited onto the MN mould, followed by centrifugation for 8 min at 4000 rpm to compact the mixture flow into the cavities. The fabrication deposition process was repeated three times for uniformity, and the residue mixture on the mould surface was recycled. Next, 2.0 g/ml of the PVP mixture was added and centrifuged three times to prepare the micro-mould reservoir. After drying at 37 °C in a vacuum desiccator overnight, the needle arrays were carefully peeled off from the mould and stored in dry conditions.

***In vivo* catalytic therapeutic assay.** Five-week-old female BALB/c nude mice (∼15 g) were purchased from SLAC for assessment of the catalytic therapeutic performance. The right flank of the leg was subcutaneously injected with 4T1 tumour cells (1×10^6^ cells/site, 100 μl of saline) to establish the 4T1 tumour model. Fifty tumour-bearing mice (tumour volume ∼80 mm^3^, 10 d after tumour inoculation) were randomly divided into five groups (n = 10 for each group) and intratumourally administered (1) blank MN, (2) HNCS-MN, (3) C-HNCS-MN, (4) F-HNCS-MN, and (5) CF-HNCS-MN. A digital calliper was used to measure the length (L) and width (W) of the tumours every 2 d for 14 d. The formulas V = L × W^2^/2 and RTI = 1– (V_n，CF-HNCS_/V_0，CF-HNCS_)/(V_n，Control_/V_0，Control_) were employed to calculate the tumour volume and the RTI rate, respectively, where V_n_ represents the tumour volume on day n. Meanwhile, five 4T1 xenografted tumour-bearing BALB/c mice were injected with (1) blank MN, (2) HNCS-MN, (3) C-HNCS-MN, (4) F-HNCS-MN, or (5) CF-HNCS-MN. At 14 d post-injection, their tumours were dissected and fixed in 10% formalin. The tumour tissues were stained with H&E, TUNEL, and Ki-67 for analysis. Mice were euthanized after the tumour volume surpassed 1000 mm^3^.

Quantitative data in this article are expressed as the mean ± standard deviation (SD). The unpaired Student’s two-sided t-test was employed to assess the signiﬁcant differences (**P* < 0.1, ***P* < 0.05, ****P* < 0.005).

**Data availability.** All data are available from the authors upon reasonable request.

**Author contributions**

X.L. and J.S. conceived and designed experiments. X.L. performed most of the experiments. S.G. participated in the cell and animal experiments. H.L. assisted with the animal experiments. H.T. helped with the electrochemical measurements. D.X. assisted with the catalyst synthesis. X.L. and J.S. analysed the experimental data and wrote the paper. All the authors discussed the results and commented on the manuscript.

**Competing financial interests**

The authors declare no competing financial interests.

**Additional information**

**Supplementary information** is available in the online version of the paper.

**Correspondence and requests for materials** should be addressed to J.S.

**Part B. Supplementary scheme**

**Supplementary Scheme 1.** Schematic of the quaternization reaction.

**Part C. Supplementary figures**


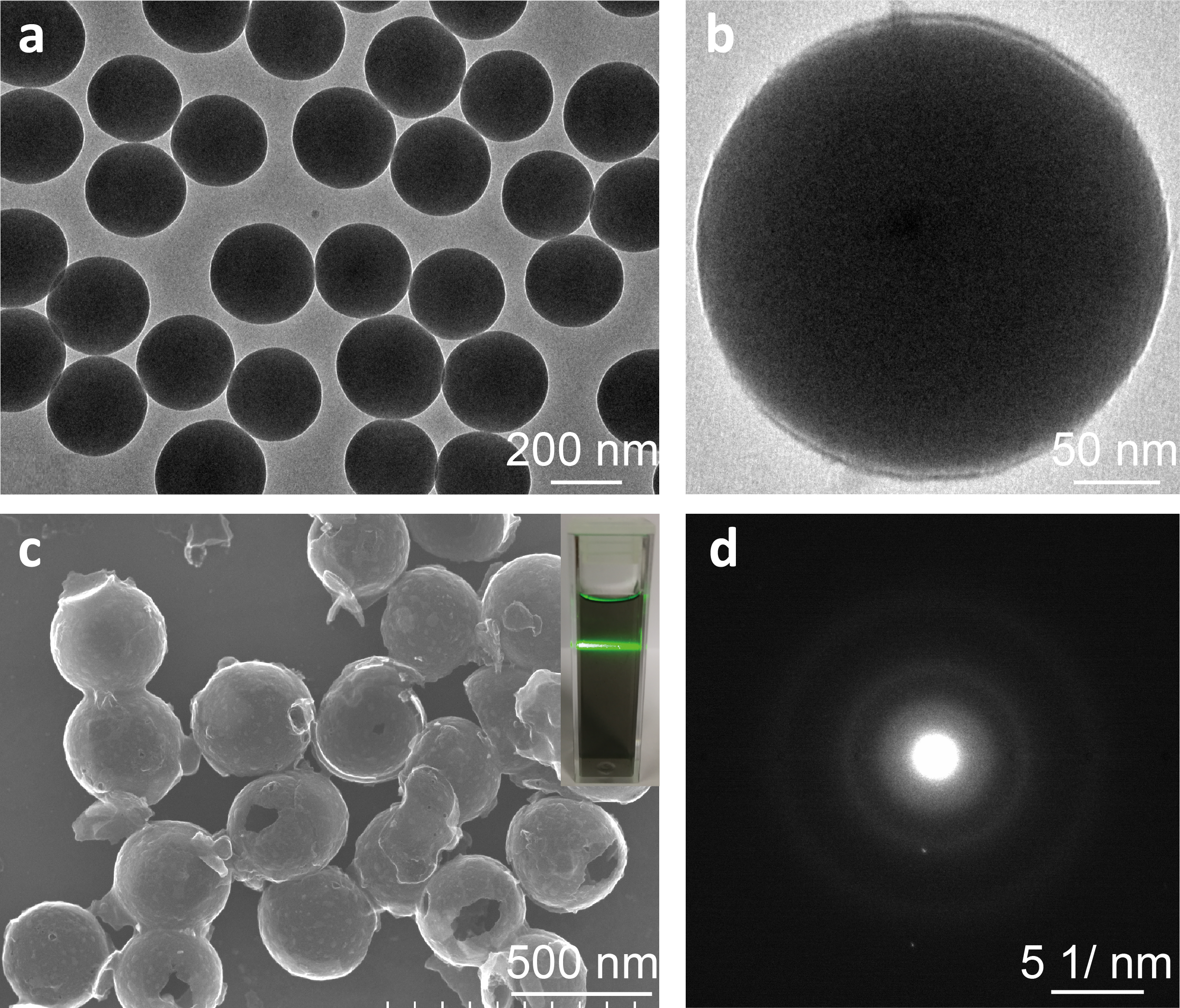


**Supplementary Figure 1.** **a**, TEM image of SiO_2_ nanoparticles. **b**, TEM image of a SiO_2_*@*Cu/Fe-TIPP/TIPP-polymer nanoparticle. **c**, SEM image of CF-HNCS. **d**, SAED pattern of CF-HNCS.

**Supplementary Figure 2. a**, XRD pattern of CF-HNCS. **b**, Raman spectra of various samples. **c**, N 1s XPS spectra of CF-HNCS. **d**, FT-IR spectra of various samples.

**Supplementary Figure 3. a**, Cu 2*p* XPS spectra of CF-HNCS and C-HNCS. **d**, Fe 2*p* XPS spectra of CF-HNCS and F-HNCS.

**Supplementary Figure 4. a**, XANES spectra at the Cu K-edge of C-HNCS and reference samples. **b**, Fourier transforms at the Cu K-edge of C-HNCS and reference samples. **c-d**, Corresponding Cu K-edge EXAFS fitting result of C-HNCS in R space (**c**) and k space (**d**). **e**, XRD pattern of C-HNCS. **f**, Proposed arrangement of Cu single sites in C-HNCS.

**Supplementary Figure 5.** **a**, XANES spectra at the Fe K-edge of F-HNCS and reference samples. **b**, Fourier transforms at the Fe K-edge of F-HNCS and reference samples. **c-d**, Corresponding Fe K-edge EXAFS fitting result of F-HNCS in R space (**c**) and k space (**d**). **e**, XRD pattern of F-HNCS. **f**, Proposed arrangement of Fe single sites in F-HNCS.


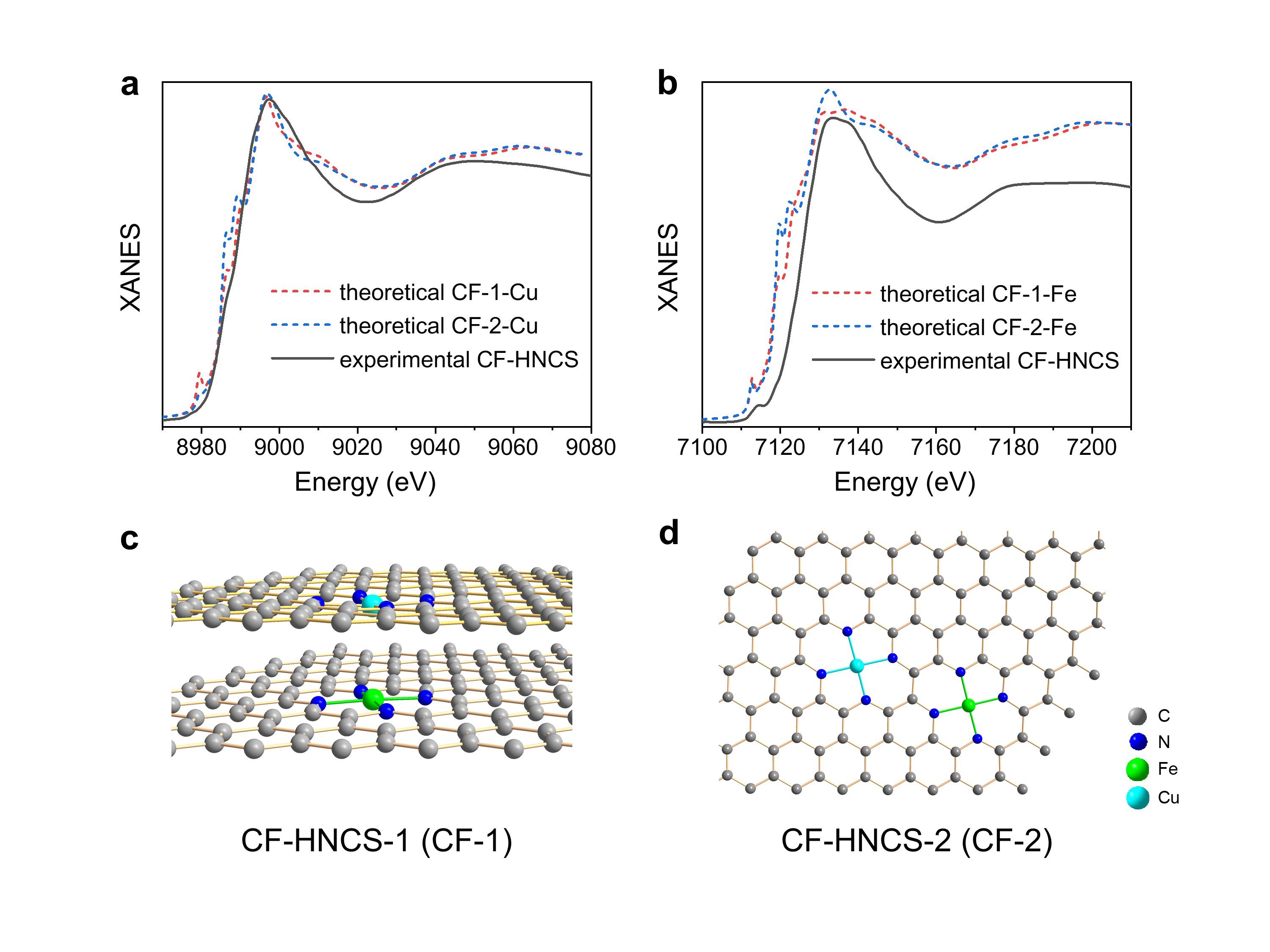


**Supplementary Figure 6. a**, Comparison between the Cu K-edge XANES experimental spectra of CF-HNCS (solid lines) and the theoretical spectra calculated based on the proposed structures (dashed lines). **b**, Comparison between the Fe K-edge XANES experimental spectra of CF-HNCS (solid lines) and the theoretical spectra calculated with the proposed structures (dashed lines). **c-d**, Proposed architectures of Cu-Fe dual sites (CF-HNCS-1, **c**) and (CF-HNCS-2, **d**).

**Supplementary Figure 7.** Oxidase-mimicking activity of CF-HNCS. **a**, Michaelis–Menten kinetics curves for CF-HNCS with TMB as a substrate. **b**, Lineweaver–Burk plots for CF-HNCS with TMB as a substrate.

**Supplementary Figure 8.** **a**, Time-dependent absorbance changes of 0.1 mM TMB in the presence of various samples in NaAc buffer solution. **b**, LSV curves of various samples in O_2_-saturated NaAc buffer solution. **c,** Linear fitting between the *v*_n_ of the chromogenic reaction at 0.1 mM TMB in NaAc buffer solution and the $\left| \overline{j_{n}} \right|$ from 0.9 to 0 V in the ORR in O_2_-saturated NaAc buffer solution.

**Supplementary Figure 9. a**, UV-vis absorption spectra of OPD after incubation with various samples for 60 min in deionized water. **b,** Fitting between the chromogenic reaction of OPD in deionized water and the $\left| \overline{j_{n}} \right|$ from 0.9 to 0 V in the ORR in deionized water.

**Supplementary Figure 10. a**, Effects of temperature on the catalytic activity of CF-HNCS. **b**, Effects of pH on the catalytic activity of CF-HNCS. (each group n = 3, bars represent mean ± s.d.)

**Supplementary Figure 11. a**, Stability measurements for CF-HNCS and PtC at the initial cycle and after 20 cycles in SBF solution. **b**, Stability measurements for CF-HNCS and PtC at the initial cycle and after 30 cycles in DMEM solution.

**Supplementary Figure 12. a**, CVs at the CF-HNCS electrodes after a continuous potentiodynamic sweep for ~50 cycles in various O_2_-saturated solutions at room temperature (~25 °C). Scan rate, 0.1 V s^−1^. **b**, ESR spectra of BMPO/•OOH at pH 4.5. **c**, Fluorescence spectra of DHE incubated with CF-HNCS with or without the addition of GSH.


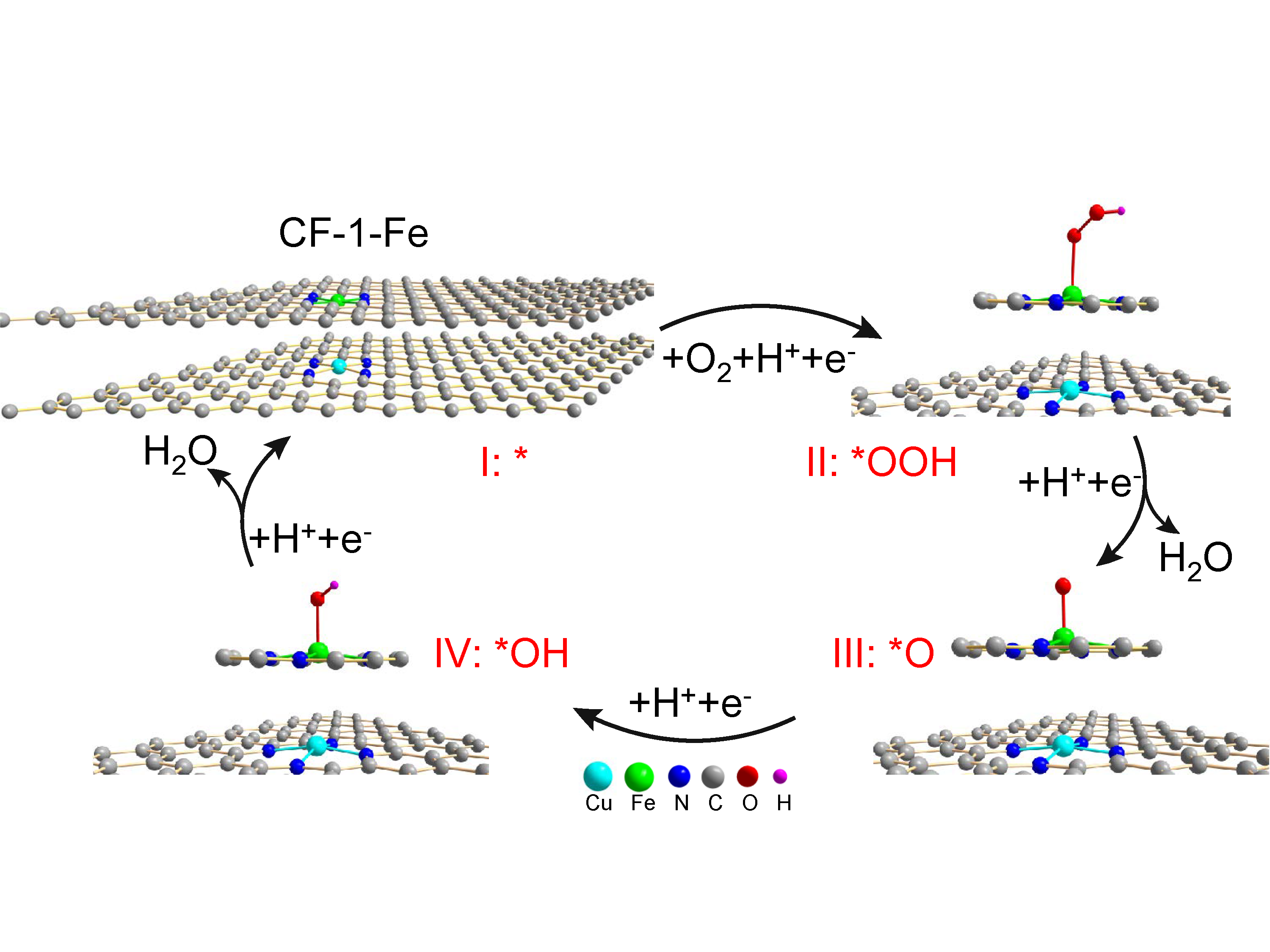


**Supplementary Figure 13.** Proposed reaction pathways of the ORR on CF-1-Fe. The grey, dark blue, baby blue, green, red, and pink balls represent the C, N, Cu, Fe, O, and H atoms, respectively.

**Supplementary Figure 14. a-d,** Free energy pathway profiles of the ORR on different active sites at pH = 1.0 (**a**), 4.5 (**b**), 6.0 (**c**), and 7.4 (**d**).

**Supplementary Figure 15**. **a**, Intracellular ROS levels investigated with a DHE fluorescent probe by flow cytometry in 4T1 cells. **b**, Bar graph showing the median DHE fluorescence intensity in the flow cytometry analysis.


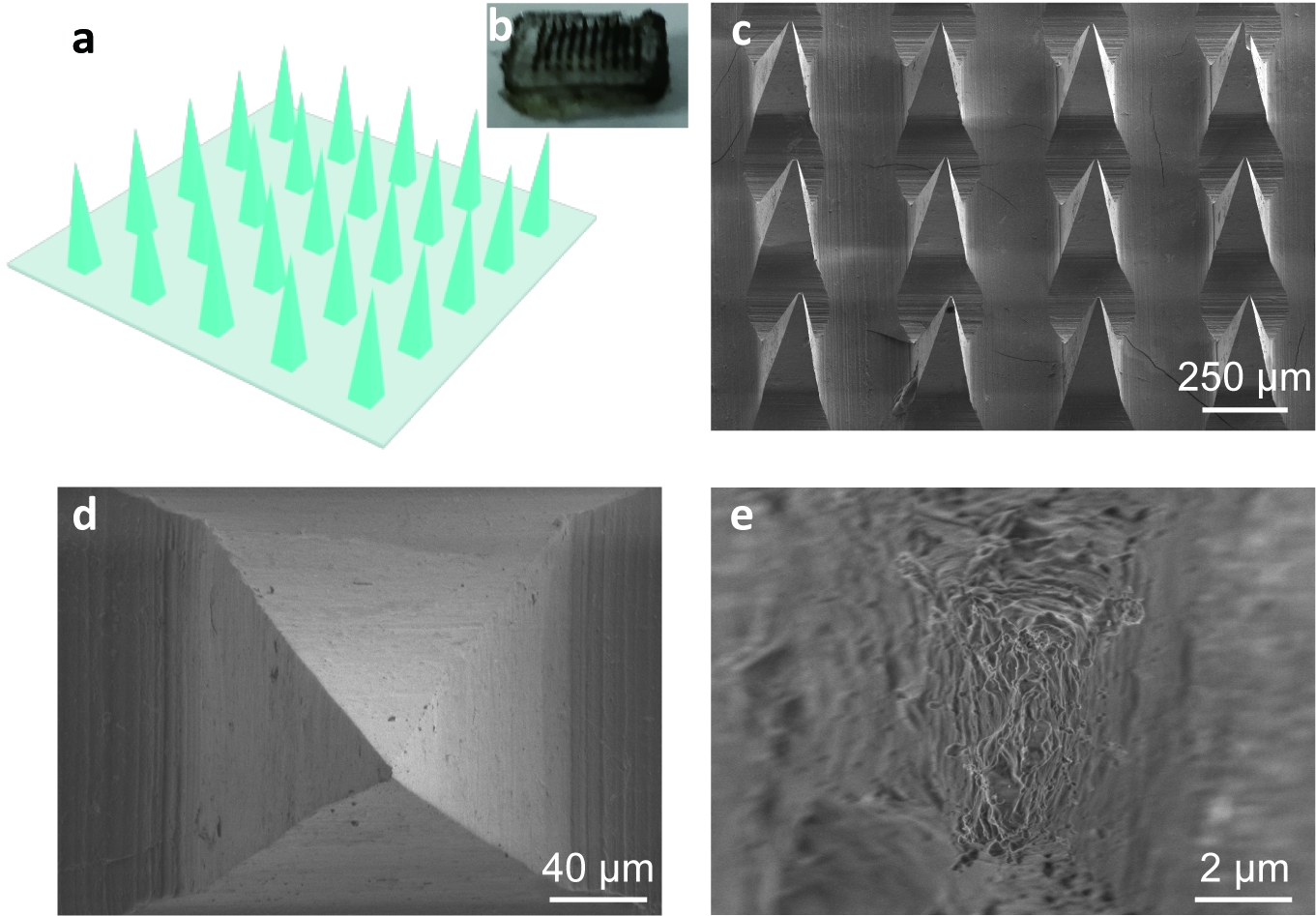


**Supplementary Figure 16. a**, Schematic illustration of the MN. **b**, Digital photograph of the MN. **c-e**, SEM images of the MN at different magnifications.

**Supplementary Figure 17.** Schematic illustration of MN administration.


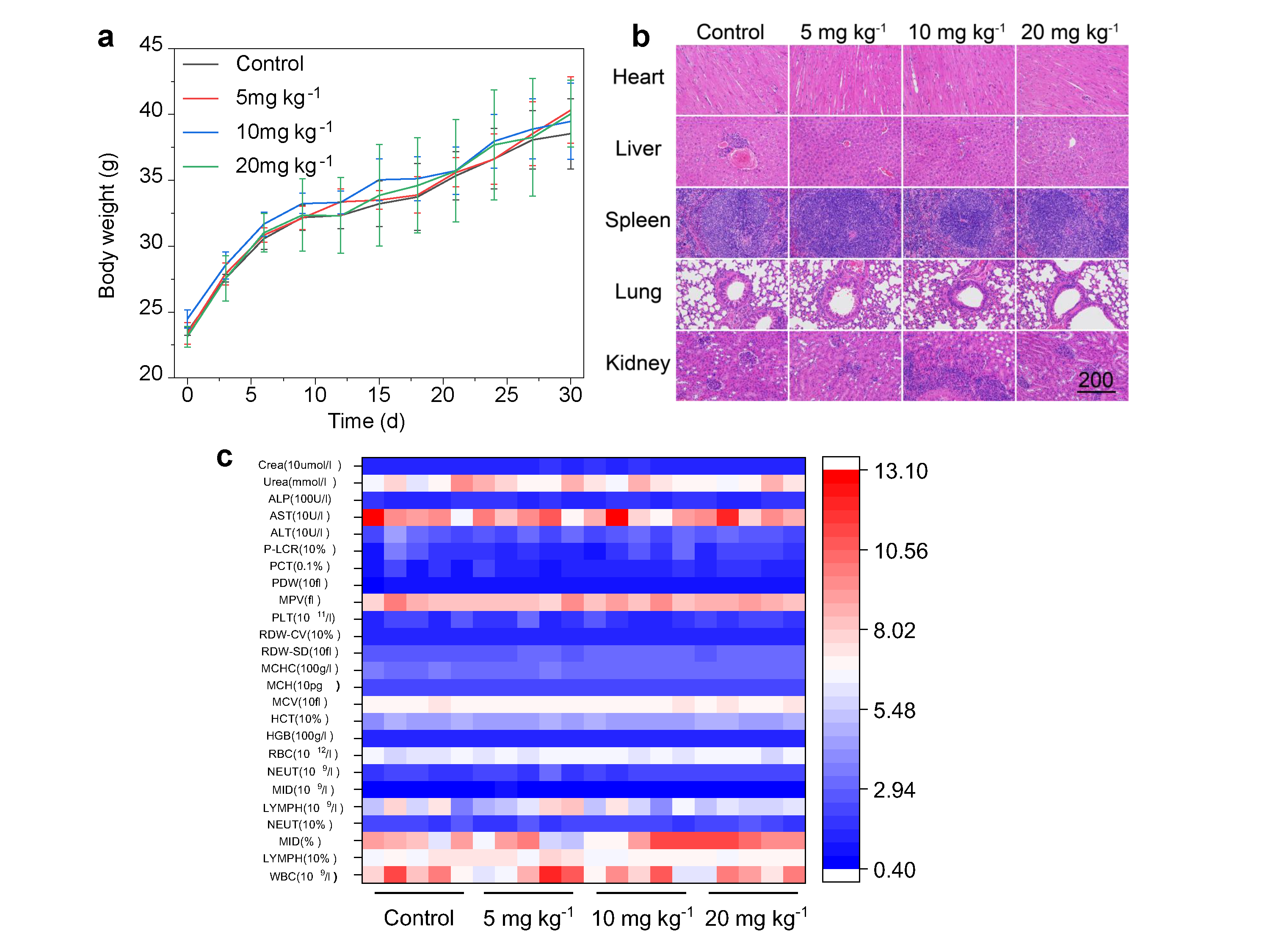


**Supplementary Figure 18. a**, Body weight changes of Kunming mice injected with the Cu-HNCS catalysts (each group n = 5, mean ± s.d.). **b**, H&E staining for cell necrosis in major organs. Scale bars: 200 μm. **c**, Levels of different serum biochemical assay and blood panel analysis parameters for mice (n = 5) after 30 d of treatment: Crea (10 μmol/L), Urea (mmol/L), ALP (100 U/L), AST (10 U/L), ALT (10 U/L), P-LCR (10%), PCT (0.1%), PDW (10 fL), MPV (fL), PLT (10^11^ /L), RDW-CV (10%), RDW-SD (10 fL), MCHC (100 g/L), MCH (10 pg), MCV (10 fL), HCT (10%), HGB (100 g/L), RBC (10^12^ /L), NEUT# (10^9^ /L), MID# (10^9^ /L), LYMPH# (10^9^ /L), NEUT (10%), MID (%), LYMPH (10%), and WBC (10^9^ /L).

**Part D. Supplementary tables**

**Supplementary Table 1.** EXAFS fitting parameters at the Cu K-edge of various samples.

| Sample | Path | C.N. | R (Å) | σ^2^×10^3^ (Å^2^) | ΔE (eV) | R factor |
| --- | --- | --- | --- | --- | --- | --- |
| Cu foil | Cu-Cu | 12* | 2.54±0.01 | 8.6±0.3 | 3.5±0.6 | 0.003 |
| C-HNCS | Cu-N | 4.0±0.6 | 1.95±0.01 | 7.1±1.3 | 0.0±2.1 | 0.011 |
| CF-HNCS | Cu-N | 4.8±0.8 | 1.96±0.01 | 7.4±1.5 | -0.3±1.7 | 0.010 |
|  | Cu-Fe | 0.9±1.1 | 2.86±0.08 | 11.7±11.1 | -0.6±12.6 |  |

*^a^N*: coordination number; *^b^R*: bond distance; *^c^σ*^2^: Debye-Waller factor; *^d^* Δ*E*_0_: inner potential correction; *R* factor: goodness of fit. * the experimental EXAFS fit of metal foil by fixing the CN as the known crystallographic value.

**Supplementary Table 2.** EXAFS fitting parameters at the Fe K-edge of various samples.

| Sample | Path | C.N. | R (Å) | σ^2^×10^3^ (Å^2^) | ΔE (eV) | R factor |
| --- | --- | --- | --- | --- | --- | --- |
| Fe foil | Fe-Fe | 8* | 2.46±0.01 | 2.8±1.1 | 6.0±1.7 | 0.003 |
|  | Fe-Fe | 6* | 2.85±0.02 | 3.4±1.9 | 6.2±3.0 |  |
| F-HNCS | Fe-N | 3.6±0.6 | 2.00±0.01 | 14.3±1.8 | 0±2.3 | 0.008 |
| CF-HNCS | Fe-N | 4.3±1.4 | 1.95±0.02 | 7.6±3.6 | -6.4±3.4 | 0.013 |
|  | Fe-Cu | 0.9±1.1 | 3.13±0.16 | 12.3±15.4 | -3.0±6.1 |  |

*^a^N*: coordination number; *^b^R*: bond distance; *^c^σ*^2^: Debye-Waller factor; *^d^* Δ*E*_0_: inner potential correction; *R* factor: goodness of fit. * the experimental EXAFS fit of metal foil by fixing the CN as the known crystallographic value.

**Supplementary Table 3.** Comparison of the kinetics based on the concentration of metal active sites in the CF-HNCS, and with the previously reported mimetic oxidase.

| **Catalysts** | **Km（**mM**）** | ***Vmax***  **（**μM s^-1^**）** | | ***Kcat***  **(**s^-1^**)** | ***Kcat/Km***  (M^-1^ s^-1^) | **U/mg** | **Refs.** |
| --- | --- | --- | --- | --- | --- | --- | --- |
| CF-HNCS | 0.21 | | 0.28 | 0.0845 | 399 | 89.86 | Our work |
| CeO_2_ NPs | 0.42 | | 1.00 | 0.0017 | 4.10 |  | *ACS Sens.* **2016**, 1: 1336-1343. |
| SO_4_^2−^/CeO_2_ NRs | 0.22 | | 4.8 | 0.0041 | 18.76 |  | *Chem. Eng. J.* **2017**, 330: 746-752. |
| Se NPs | 8.3 | | 0.51 | 0.00067 | 0.081 |  | *J.Nanopart. Res.* **2016**, 18: 74. |
| NiCo_2_O_4_ MS | 0.13 | | 0.10 | 0.0004 | 3.09 |  | *Anal. Chim. Acta* **2017**, 951: 124-132. |
| Fe single atom nanozyme | 0.13 | | 0.0225 | 0.021 | 162 |  | *Chem. Commun*., **2019**, 55: 2285-2288 |
| Fe-N/C SACs | 0.94 | | 0.598 | 0.038 | 40 |  | *Sensors & Actuators: B. Chemical* **2020**, 305: 127511 |

**Part E. Supplementary Discussions**

**Discussion S1.** **Structural characterization by SAED, XRD, Raman spectroscopy, XPS and FT-IR spectroscopy.**

The diffuse halo in the selected-area electron diffraction (SAED) pattern confirms the poor crystalline nature of CF-HNCS (Figure S3d). The XRD pattern of CF-HNCS shows a broadened peak corresponding to the (002) plane at approximately 24.8° with a d-spacing of ∼0.356 nm (Figure S2a), which is very close to the HR-TEM result and further demonstrates the relatively low level of crystallization of the carbon matrix. No characteristic peaks of any copper- or iron-based crystallized phases were observed, indicating the absence of clustered or grained copper and iron species.

Figure S2b demonstrates two Raman peaks at approximately 1337 and 1596 cm^–1^, which originate from the well-defined D band induced by defects and the G band induced by graphitic carbon, respectively. The *I_D_*/*I_G_* values of the CF-HNCS, C-HNCS, F-HNCS, and HNCS catalysts were calculated to be 0.98, 0.98, 0.97, and 0.91, respectively, which means that the topological defects slightly increase due to metal doping ^[10]^. This suggests that the metal sites, rather than topological defects, serve as the active sites for catalytic reactions in our experiments. Moreover, no impurity peaks, such as those of crystallized Cu and Fe metals, can be observed in the Raman spectrum ^[11]^.

XPS was employed to study the bonding configurations of nitrogen atoms in CF-HNCS. The N 1s XPS spectra can be deconvoluted into four peaks corresponding to pyridinic N (398.6 eV), pyrrolic N (400.0 eV), graphitic N (401.2 eV), and oxidized N (403.2 eV) by least-squares fitting (Figure S2c) ^[12]^. The calculated percentages of pyridinic N, pyrrolic N, graphitic N and oxidized N species are 40.7%, 4.6%, 50.7% and 4.0%, respectively. As is evident, the pyridinic N anchors the metal atoms to form Cu-N_x_ and Fe-N_x_ structures, agreeing with the analysis in previous reports ^[1]^.

Fourier transform infrared (FT-IR) spectroscopy images show the absorption bands of the C-O bending, C=O stretching and O-H stretching vibrations at 1394 cm^–1^, 1632 cm^–1^ and 3425 cm^–1^, respectively (Figure S2d). Biomedical applications will benefit from particle surfaces with large amounts of carboxyl groups and hydroxyl groups.

**Discussion S2. *In vivo* toxicology evaluation analysis.**

The *in vivo* chronic biosafety (30 days) was monitored by injecting Cu-HNCS catalysts into the right leg of SPF-level healthy Kunming mice. During the evaluation, no significant difference in mouse body weights was observed in the different groups (Figure S18a). Furthermore, the blood biochemical parameters and regular haematological indexes were found to be normal compared to those of the control group, suggesting that Cu-HNCS caused little hepatotoxicity, nephrotoxicity, infection, or inflammation (Figure S18b). In addition, the intact functional units and cellular morphologies in the histological H&E staining of major organs are similar in different groups, further proving the excellent biosafety for *in vivo* therapeutic application (Figure S18c).

**Part F. Supplementary references**

[1] Han Y, Wang Y G, Chen W, *et al*. Hollow N-Doped Carbon Spheres with Isolated Cobalt Single Atomic Sites: Superior Electrocatalysts for Oxygen Reduction. *J Am Chem Soc* 2017; **139**: 17269-17272.

[2] Wang Y, Zhao H, Li X, *et al*. A durable luminescent ionic polymer for rapid detection and efficient removal of toxic Cr_2_O_7_^2−^. *J Mater Chem A* 2016; **4**: 12554-12560.

[3] Yu H-S, W X-J, Li J, *et al*. The XAFS beamline of SSRF. *Nucl Sci Tech*. 2015; **26**: 50102-050102.

[4] Ravel B, Newville M J J O S R. ATHENA, ARTEMIS, HEPHAESTUS: data analysis for X-ray absorption spectroscopy using IFEFFIT. *J Synchrotron Radiat* 2005; **12**: 537-541.

[5] Funke H, Chukalina M, Scheinost A C J J O S R. A new FEFF-based wavelet for EXAFS data analysis. *J Synchrotron Radiat* 2007; **14**: 426-432.

[6] Kresse G, Furthmüller J J P R B. Efficient iterative schemes for ab initio total-energy calculations using a plane-wave basis set. *Physical Review B: Condensed Matter and Materials Physics* 1996; **54**: 11169.

[7] Kresse G, Furthmüller J J C M S. Efficiency of ab-initio total energy calculations for metals and semiconductors using a plane-wave basis set. *Comput Mater Sci* 1996; **6**: 15-50.

[8] Perdew J P, Burke K, Ernzerhof M J P R L. Generalized gradient approximation made simple. *Phys Rev Lett* 1996; **77**: 3865.

[9] Grimme S, Antony J, Ehrlich S, *et al*. A consistent and accurate ab initio parametrization of density functional dispersion correction (DFT-D) for the 94 elements H-Pu. *J Chem Phys* 2010; **132**: 154104.

[10] Jia Y, Zhang L, Zhuang L, *et al*. Identification of active sites for acidic oxygen reduction on carbon catalysts with and without nitrogen doping. *Nat Catal* 2019; **2**: 688-695.

[11] Yang Z, Chen B, Chen W, *et al*. Directly transforming copper (I) oxide bulk into isolated single-atom copper sites catalyst through gas-transport approach. *Nat Commun* 2019; **10**: 3734.

[12] Guo D, Shibuya R, Akiba C, *et al*. Active sites of nitrogen-doped carbon materials for oxygen reduction reaction clarified using model catalysts. *Science* 2016; **351**: 361-365.
